# Prognostic accuracy of qSOFA score, SIRS criteria, and EWSs for in-hospital mortality among adult patients presenting with suspected infection to the emergency department (PASSEM) *Multicenter prospective external validation cohort study protocol*

**DOI:** 10.1371/journal.pone.0281208

**Published:** 2024-01-17

**Authors:** Abdullah M. Algarni, Musa S. Alfaifi, Ali A. Al Bshabshe, Othman M. Omair, Mohammed A. Alsultan, Hasan M. Alzahrani, Hadi E. Alali, Abdullah A. Alsabaani, Ali M. Alqarni, Salah A. Alghanem, Bandar S. Al Mufareh, Ayesha M. Almemari, Abdulrahman A. Sindi, Ibrahim U. Ozturan, Abdullah A. Alhadhira, Asaad S. Shujaa, Abdullah H. Alotaibi, Moosa M. Awladthani, Ahmed A. Alsaad, Abdullah A. Almarshed, AlHanouv M. AlQahtani, Tim R. Harris, Bader A. Alyahya, Saad A. Assiri, Feras H. Abuzeyad, Sara N. Kazim, Abdulrahman A. Al-Fares, Faisal Y. Almazroua, Naif T. Marzook, Abdullah A. Basri, Abdallah M. Elsafti, Abdulaziz S. Alalshaikh, Cansu A. Özturan, Yousef I. Alawad, Awad AlOmari, Malek A. Alkhateeb, Moonis M. Farooq, Latifa Ali AlMutairi, Muneera M. Alasfour, Mohammad I. Al Haber, Umma-Kulthum A. Umar, Nidal H. Bokhary, Saeed F. Alqahtani, Abdulrhman Almutairi, Hisham F. Alyahya, Wejdan S. Alzahrani, Fawziah Alsalmi, Abdulmajeed M. Omair, Faysal M. Alasmari, Sultan Y. Alfifi, Mohammed S. Al-Nujimi, Farid Foroutan

**Affiliations:** 1 Family Medicine Department, Aseer Central Hospital, Abha, Saudi Arabia; 2 Emergency Medicine Department, Armed Forces Hospital Southern Region, Khamis Mushait, Saudi Arabia; 3 College of Medicine, King Khalid University, Abha, Saudi Arabia; 4 Emergency Medicine Department, Aseer Central Hospital, Abha, Saudi Arabia; 5 Saudi Commission for Health Specialities, Riyadh, Saudi Arabia; 6 Radiology Department, Prince Mashary Bin Saud Hospital, Belgraishi, Saudi Arabia; 7 Emergency Medicine Department, Bahrain Defence Force Hospital, Al Riffa, Bahrain; 8 Emergency Medicine Department, Royal Commission Hospital in Jubail, Jubail, Saudi Arabia; 9 Emergency Medicine Department, Shaikh Shakhbout Medical City, Abu Dhabi, United Arab Emirates; 10 College of Medicine, King Abdulaziz University, Jeddah, Saudi Arabia; 11 Kocaeli University, Faculty of Medicine, Emergency Medicine Department, Kocaeli, Turkey; 12 Emergency Medicine Department, Johns Hopkins Aramco Healthcare, Dhahran, Saudi Arabia; 13 Emergency Medicine Department, King Abdullah University Hospital, Riyadh, Saudi Arabia; 14 Critical Care Department, Armed Forces Hospital Oman, Muscat, Oman; 15 Emergency Medicine Department, King Fahad Specialist Hospital, Dammam, Saudi Arabia; 16 Emergency Medicine Department, King Fahad Medical City, Riyadh, Saudi Arabia; 17 Emergency Medicine Department, North Medical Tower Hospital, Arar, Saudi Arabia; 18 Emergency Medicine Department, Hamad Medical Corporation, Doha, Qatar; 19 College of Medicine, King Saud University, Riyadh, Saudi Arabia; 20 Emergency Medicine Department, Sulaiman Al Habib Medical Group, Riyadh, Saudi Arabia; 21 Emergency Medicine Department, King Hamad University Hospital, Muharraq, Bahrain; 22 Emergency Medicine Department, Rashid Hospital, Dubai, United Arab Emirates; 23 Critical Care Department, Al Amiri hospital, Kuwait City, Kuwait; 24 Emergency Medicine Department, King Saud Medical City, Riyadh, Saudi Arabia; 25 Emergency Medicine Department, King Fahad Armed Forces Hospital, Jeddah, Saudi Arabia; 26 Emergency Medicine Department, Gölcük Necati Çelik State Hospital, Gölcük, Kocaeli, Turkey; 27 Emergency Medicine Administration, King Fahad Medical City, Riyadh, Saudi Arabia; 28 Critical Care Department, Sulaiman Al Habib Medical Group, Riyadh, Saudi Arabia; 29 Emergency Medicine Department, Al Amiri Hospital, Kuwait City, Kuwait; 30 Health Research Methods, Evidence and Impact, McMaster University, Hamilton, Ontario, Canada; University of Science and Technology of Fujairah, YEMEN

## Abstract

**Background:**

Early identification of a patient with infection who may develop sepsis is of utmost importance. Unfortunately, this remains elusive because no single clinical measure or test can reflect complex pathophysiological changes in patients with sepsis. However, multiple clinical and laboratory parameters indicate impending sepsis and organ dysfunction. Screening tools using these parameters can help identify the condition, such as SIRS, quick SOFA (qSOFA), National Early Warning Score (NEWS), or Modified Early Warning Score (MEWS). We aim to externally validate qSOFA, SIRS, and NEWS/NEWS2/MEWS for in-hospital mortality among adult patients with suspected infection who presenting to the emergency department.

**Methods and analysis:**

PASSEM study is an international prospective external validation cohort study. For 9 months, each participating center will recruit consecutive adult patients who visited the emergency departments with suspected infection and are planned for hospitalization. We will collect patients’ demographics, vital signs measured in the triage, initial white blood cell count, and variables required to calculate Charlson Comorbidities Index; and follow patients for 90 days since their inclusion in the study. The primary outcome will be 30-days in-hospital mortality. The secondary outcome will be intensive care unit (ICU) admission, prolonged stay in the ICU (i.e., ≥72 hours), and 30- as well as 90-days all-cause mortality. The study started in December 2021 and planned to enroll 2851 patients to reach 200 in-hospital death. The sample size is adaptive and will be adjusted based on prespecified consecutive interim analyses.

**Discussion:**

PASSEM study will be the first international multicenter prospective cohort study that designated to externally validate qSOFA score, SIRS criteria, and EWSs for in-hospital mortality among adult patients with suspected infection presenting to the ED in the Middle East region.

**Study registration:**

The study is registered at ClinicalTrials.gov (NCT05172479).

## Introduction

Over the past decade, there has been continued focus on sepsis as a prevalent condition that accounts for 10% of admissions to intensive care units (ICUs) and is associated with a 10–20% in-hospital mortality rate [1–5]. Standardized protocols and physician awareness have significantly improved survival, but mortality rates remain high between 20% and 36%, with ~270,000 deaths annually in the United States [6–8]. It has been estimated that 80% of sepsis cases are identified and treated in the emergency department (ED), and the remainder develop sepsis during hospitalization with other conditions [7].

In 2016, the Society of Critical Care Medicine/European Society of Intensive Care Medicine (SCCM/ESICM) task force redefined sepsis based on organ dysfunction and mortality prediction [9–11]. Sepsis now is defined as life-threatening organ dysfunction caused by dysregulated host response to infection. This definition emphasizes the complexity of the disease that cannot be explained by infection or body response alone. Acute change in Sequential Organ Failure Assessment (SOFA) score ≥2 indicates sepsis-related organ dysfunction and is associated with in-hospital mortality. Systemic Inflammatory Response Syndrome (SIRS) and “severe sepsis” terms were omitted from the most recent definition. SIRS has been criticized for its poor specificity, while “severe sepsis” may underestimate sepsis’s seriousness. A subset of patients may develop septic shock with underlying profound organ dysfunction and excess mortality. Clinically, septic shock is defined as persistent hypotension requiring vasopressors to maintain mean arterial pressure (MAP) ≥ 65 mm Hg and serum lactate level ≥ 2 mmol/L (18 mg/dL) despite adequate volume resuscitation.

Early identification of a patient with infection who may develop sepsis is of utmost importance [12]. Unfortunately, this remains elusive because no single clinical measure or test can reflect the complex pathophysiological changes in patients with sepsis. However, multiple clinical and laboratory parameters indicate impending sepsis and organ dysfunction. Screening tools using these parameters can help identify the condition, such as SIRS, quick SOFA (qSOFA), National Early Warning Score (NEWS), or Modified Early Warning Score (MEWS) (Tables 1 and 2) [13].

**Table 1:** Component of qSOFA score and SIRS criteria.

Variable	qSOFA		SIRS	
	*Cut-off*	*Points*	*Cut-off*	*Points*
Altered mental status (GCS <15)	Yes	1	—	—
Heart rate (beats/min)	—	—	>90	1
Respiratory rate (breaths/min)	≥22	1	>20	1
Systolic blood pressure (mm Hg)	≤100	1	—	—
Temperature (°C)	—	—	<36 or >38	1
White blood cells count (x10^9^/μL)	—	—	<4 or >12 or >10% bands	1
	**Maximum score**	**3**	**Maximum score**	**4**
	**Positive cut-off value**	**≥2**	**Positive cut-off value**	**≥2**

***GCS*:** Glasgow Coma Scale; ***qSOFA*:** quick Sequential Organ Failure Assessment; ***SIRS*:** Systemic inflammatory response syndrome.

**Table 2:** Components of NEWS, NEWS2, and MEWS.

Variable	NEWS		NEWS2		MEWS	
	*Cut-off*	*Points*	*Cut-off*	*Points*	*Cut-off*	*Points*
AVPU	Alert	0	Alert	0	Alert	0
	VPU	3	CVPU*	3	React to voice (V)	1
	—	—	—	—	React to pain (P)	2
	—	—	—	—	Unresponsive (U)	3
HR (beats/min)	51–90	0	51–90	0	51–100	0
	91–110; or 41–50	1	91–110; or 41–50	1	41–50 or 101–110	1
	111–130	2	111–130	2	<40 or 111–129	2
	≤40 or ≥131	3	≤40 or ≥131	3	≥130	3
O_2_Sat (%)	≥96	0	≥96^†^	0	—	—
	94–95	1	94–95	1	—	—
	92–93	2	92–93	2	—	—
	≤91	3	≤91	3	—	—
Oxygen supp.	No	0	No	0	—	—
	Yes	2	Yes	2	—	—
RR (breaths/min)	12–20	0	12–20	0	9–14	0
	9–11	1	9–11	1	15–20	1
	21–24	2	21–24	2	<9 or 21–29	2
	≤8 or ≥25	3	≤8 or ≥25	3	≥30	3
SBP (mm Hg)	111–219	0	111–219	0	101–199	0
	101–110	1	101–110	1	81–100	1
	91–100	2	91–100	2	71–80 or ≥200	2
	≤90 or ≥220	3	≤90 or ≥220	3	≤70	3
Temperature (°C)	36.1–38	0	36.1–38	0	35–38.4	0
	35.1–36 or 38.1–39	1	35.1–36 or 38.1–39	1	<35 or ≥38.5	2
	≥39.1	2	≥39.1	2	—	—
	≤35	3	≤35	3	—	—
	**Maximum score**	20	**Maximum score**	20	**Maximum score**	14
	**Positive cut-off value**	≥5	**Positive cut-off value**	≥5	**Positive cut-off value**	≥5

***AVPU*:** Alert, verbal, pain, or unresponsive; ***HR*:** Heart rate; ***NEWS*:** National early warning score; ***NEWS2*:** National early warning score 2; ***MEWS*:** Modified early warning score; ***O***_***2***_***Sat*:** Oxygen saturation; ***RR*:** Respiratory rate; ***SBP*:** Systolic blood pressure.

*—Level of consciousness and new confusion (‘C’), thus AVPU becomes ACVPU, where C represents new confusion.

†—NEWS2 has a dedicated section (SpO_2_ Scale 2) for use in patients with hypercapnic respiratory failure who have clinically recommended oxygen saturation of 88–92%.

The 2016 SCCM/ESICM task force recommended using qSOFA [11], while the 2021 Surviving Sepsis Campaign strongly recommended against its use compared with SIRS, NEWS, or MEWS as a single screening tool for sepsis or septic shock [14].

Multiple studies have assessed qSOFA, SIRS, and EWSs validity in ED and showed conflicting results [15–21]. One systemic review compared qSOFA and EWSs (NEWS/Modified EWS [MEWS]) for predicting mortality and ICU admission when applied in the ED [13]. None of the eligible studies included NEWS2; and the authors of the review could not perform a meta-analysis due to marked heterogeneity in patient selection, definition of infection, outcomes, and settings. Moreover, studies have calculated the scores at different times. NEWS appeared more sensitive than qSOFA for predicting ICU admission and mortality at the commonly used thresholds (i.e., ≥2 for SIRS and qSOFA; ≥5 for NEWS, NEWS2, and MEWS), whereas qSOFA was more specific [13]. This correlates with previous criticisms of qSOFA, which have low sensitivity for early risk assessment [18–21].

We hypothesized that qSOFA has greater prognostic accuracy than SIRS and EWSs (NEWS/NEWS2/MEWS), and subsequently, aimed to reject the null hypothesis that all these predictive models have the same prognostic accuracy. Accordingly, we developed a protocol for a prognostic study to determine whether qSOFA has higher predictive performance for relevant clinical outcomes in adult patients with infection presenting to the ED. The primary outcome of this study is 30-days in-hospital mortality, and the secondary outcomes are ICU admission, prolonged ICU admission (i.e., ≥72 hours), 30- as well as 90-days all-cause mortality.

## Methods and analysis

### Study design and setting

**Fig 1:**
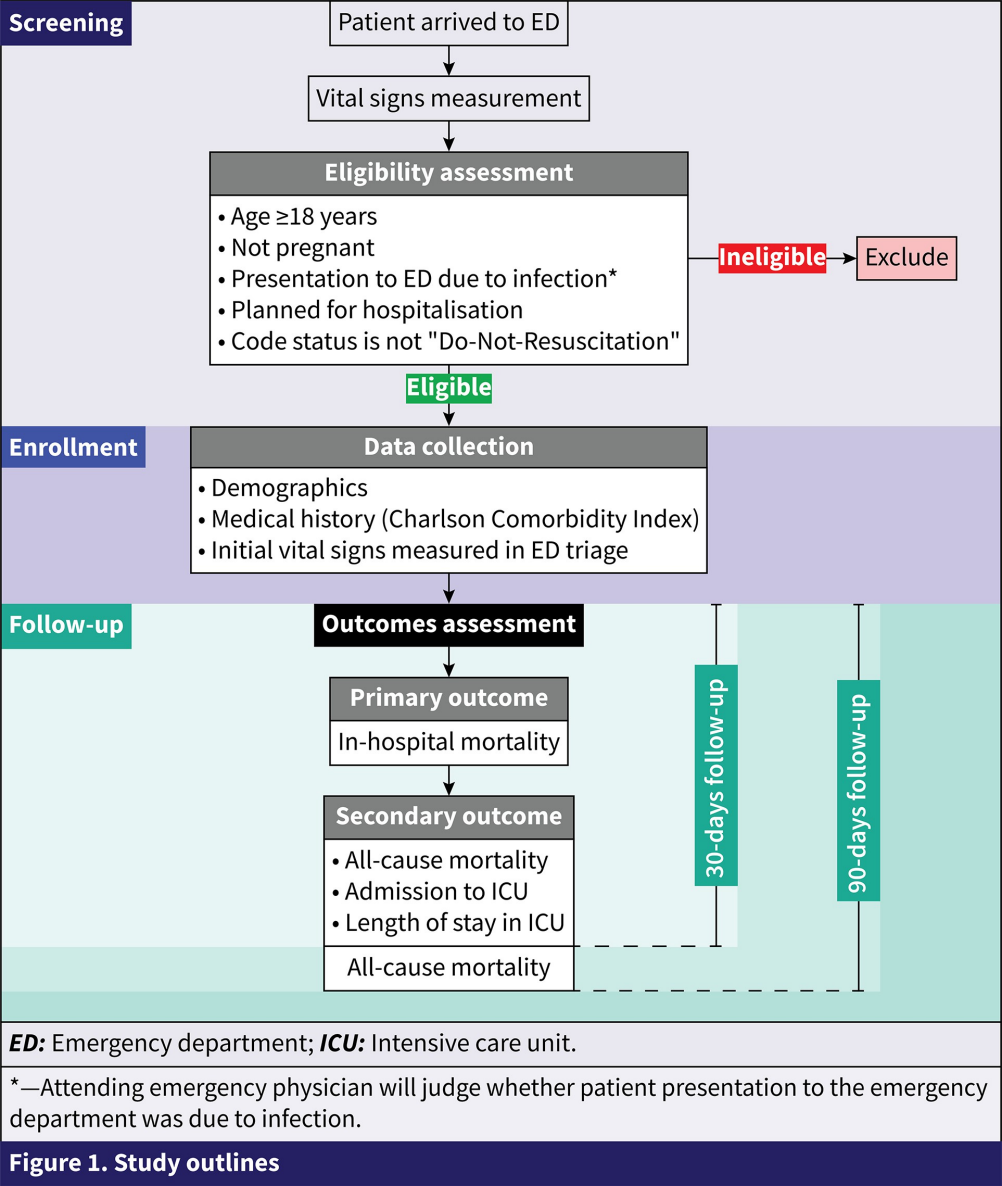
Study outlines. *—Attending emergency physician will judge whether patient presentation to the emergency department was due to infection. ***ED***: Emergency department; ***ICU***: Intensive care unit.

This protocol describes a multicenter, prospective observational cohort study evaluating the prognostic accuracy of qSOFA score, SIRS criteria, and EWSs (NEWS/NEWS2/MEWS) for in-hospital mortality among adult patients presenting to the ED with suspected infection (NCT05172479). The study’s outlines are shown in Fig 1. The study duration is 12 months per center (9 months for recruitment and 3 months for follow-up). Recruiting centers and recruitment status are shown in Table 3 (July 2023).

**Table 3:** PASSEM study centers.

Country	City	Hospital	Status upon publication
Bahrain	Al Riffa	Bahrain Defence Force Hospital	Completed
	Muharraq	King Hamad University Hospital	Completed
Kuwait	Kuwait	Al-Amiri Hospital	Completed
Oman	Muscat	Armed Forces Hospital	Completed
Qatar	Doha	Hamad General Hospital	Completed
Saudi Arabia	Arar	North Medical Tower Hospital	Active, not recruiting
	Aseer Province	Aseer Central Hospital, Abha	Completed
		Armed Forces Hospital Southern Region–Khamis Mushait	Active, not recruiting
	Eastern Province	Dr. Sulaiman Al Habib Hospitals–Khobar	Completed
		Johns Hopkins Aramco Healthcare	Completed
		King Fahad Specialist Hospital	Active, not recruiting
		Royal Commission Hospital in Jubail	Completed
	Jeddah	King Abdulaziz University Hospital	Completed
		King Fahad Armed Forces Hospital	Active, not recruiting
	Qassim Province	Dr. Sulaiman Al Habib Hospitals–Qassim	Completed
	Riyadh	Dr. Sulaiman Al Habib Hospitals–Riyadh	Completed
		King Abdullah bin Abdulaziz University Hospital	Completed
		King Fahad Medical City	Completed
		King Khalid University Hospital	Completed
		King Saud Medical City	Completed
Turkey	Kocaeli Province	Kocaeli University Hospital	Completed
United Arab Emirates	Abu Dhabi	Shaikh Shakhbout Medical City	Completed
	Dubai	Rashid Hospital	Completed

### Diagnosis of infection

A presumptive diagnosis of infection will be judged based on the opinion of the ED physician upon the initial patient presentation. If required, two experts from each recruiting center will ascertain the diagnosis of infection on the 30^th^ day since inclusion to the study. Evidence of infection will be sought by analyzing the patient’s clinical, microbiological, and radiological data. Evidence of infection would be determined by either positive culture, other microbiological techniques (e.g., serological, or molecular), or radiological findings. If all of these evidence measures were equivocal, clinical context will be used to confirm the presence of infection. In cases of disagreement, consensus will be sought between the two experts. In all cases, the diagnosis of infection will be blinded to the output of the prediction models and the outcomes of patients.

### Study population

#### Inclusion criteria

PSSEM study will enroll all consecutive adult patients (age ≥18 years) presenting to the ED with suspected infection who are planned for hospitalization (Box 1).

**Box 1. Eligibility criteria**

**Inclusion criteria**

▶ Adult patient (ages ≥18 years).
▶ Suspected infection (based on the opinion of the emergency physician).
▶ Planned for hospitalization.

**Exclusion criteria**

▶ Presentation to ED is not due to infection (e.g., autoimmune diseases, myocardial infarction, stroke, venous thromboembolism, trauma, intoxication … etc.).
▶ Pregnancy.
▶ Transferred from another hospitals.
▶ Code status is "Do-Not-Resuscitate" (DNR).
▶ Elective admission to the hospital (i.e., not through emergency department).
▶ Initial diagnosis of infection in the ED was not confirmed after finishing of the recruitment and follow-up phase.

#### Exclusion criteria

We will exclude patients who present to the ED due to non-infectious causes (e.g., autoimmune diseases, myocardial infarction, trauma, …etc.), pregnant woman, those who are transferred from other hospitals, or with “Do-Not-Resuscitate” (DNR) code status. Patients whose initial diagnosis of infection in the ED was not confirmed after the recruitment and follow-up will also be excluded (Box 1).

### PASSEM study versus original derivation cohorts

The key characteristics of PASSEM Study and the original derivation cohorts of qSOFA, SIRS, and EWSs that will be assessed are shown in Table 4.

**Table 4:** Characteristics of PASSEM study and the original development cohorts of qSOFA score, SIRS criteria, NEWS/NEWS2, and MEWS.

Characteristic	PASSEM (n = 2,851)	qSOFA (n = 1,309,025)	SIRS (n = 519)	NEWS/NEWS2 (n = 35,585)	MEWS (n = 709)
**Data collection period**	2021–2022	2010–2012	1992	2006–2008	2000
**Study design**	Prospective cohort	Retrospective cohort	Prospective cohort	Retrospective cohort	Prospective cohort
**Setting**	30 EDs across 7 countries	12 community and academic hospitals in southwestern Pennsylvania (ED, hospital ward, and ICU)	42 ICUs in 40 US hospitals	MAU at Portsmouth hospitals NHS Trust, UK	MAU at District General Hospital (DGH), UK
**Definition of infection**	Based on opinion of attending ED physician	Combination of body fluid culture and nonprophylactic antibiotic administration in the EHR	NA	NA	NA
**Inclusion criteria**	Adult patients (age ≥18 yrs.) with suspected infection who presented to the ED and planned for hospitalization	Adult patients (age ≥18 yrs.) with suspected infection	Patients with sepsis who lack a clear source of infection	All general medical emergency patients aged ≥16 yrs., except for those transferred directly to critical care areas of the hospital	All medical emergency admissions admitted to the MAU
**Primary outcome**	30-days in-hospital mortality	In-hospital mortality	24-hours in-hospital mortality	24-hours in-hospital mortality	HDU or ICU admission, attendance of the cardiac arrest team at a cardiorespiratory emergency and death at 60 days
**Time window for measuring variables**	Initial presentation (at triage)	From 48 hrs. before to 24 hrs. after the onset of infection	Upon admission to ICU	NA	Twice daily for up to 5 days

***ED*:** Emergency department; ***EHR*:** Electronic health record; ***HDU*:** High dependency unit; ***ICU*:** Intensive care unit; ***MAU*:** Medical admission unit; ***NA*:** Not available; ***NHS*:** National Health Services; ***UK*:** United Kingdom; ***US*:** United States.

### Study flow chart

The study’s procedures and assessments are shown in Table 5. Patients will undergo 4 phases: screening (Time_-1_ [T_-1_], 1–2 days), enrolment (T_0_), and in-hospital (T_1_, maximum 30 days after T_0_), and out-hospital follow-up (T_2_, maximum 90 days after T_0_).

**Table 5:** Study’s procedures and assessments.

Study components	Phases			
	Screening	Enrolment	In hospital F/U	Out hospital F/U
**Eligibility screening**	X			
**Data collection**				
Demographics, medical history		X		
Physical examination/vital signs		X		
Blood investigations (WBCs count)		X		
**Primary outcome evaluation**				
In-hospital mortality (within 30 days)			X	
**Secondary outcomes evaluation**				
1. ICU admission			X	
2. ICU length of stay			X	
3. All-cause mortality (within 30 days)				X
4. All-cause mortality (within 90 days)				X

***F/U*:** Follow-up; ***WBCs*:** White blood cells

#### Screening and enrolment phases

A staff member will screen patients for eligibility and check their measured vital signs once they arrive at the ED (triage) and the investigator will enroll potentially eligible patients (i.e., age ≥18 years, with suspected infection, and planned for hospitalization). First, a web-based electronic data capture system (EDC) will assign each patient to a participant number in ascending order. Then, the investigator will collect and enter the patient’s initial data (demographics, contact information, Charlson Comorbidity Index (CCI) components, and variables required for qSOFA, SIRS, and EWS scores calculation) in an electronic case report form (eCRF) (see online supplementary materials). If the patient is not eligible, we will close the patient record in the EDC and clarify the cause of exclusion.

#### In-hospital follow-up

Once enrolment is completed, the in-hospital follow-up phase will start (T_1_, maximum 30 days after T_0_) (Table 5). Study team will monitor hospitalized patients’ status (i.e., death, alive and either discharged, transferred to another hospital, or still hospitalized) by consulting their specific medical registration number (MRN) in the recruiting center.

#### Out-hospital follow-up

This phase starts if the patient is discharged from the hospital or 30-days have passed since inclusion to the study (whenever earlier; T_2_, maximum 90 days after T_0_) (Table 5). We will determine their status via telephone contact. We will also evaluate hospitalized patients’ situations by consulting their MRN in the recruiting center. We will consider a patient lost to follow-up if we cannot reach them via telephone contact by the end of this phase.

### Study outcome

The primary outcome of this study is 30-days in-hospital mortality. Secondary outcomes include ICU admission (within 30-days), ICU length of stay, and all-cause mortality within 30 and 90 days.

### Predictors

Lead investigator in each center will extract the demographics, components of CCI, vital signs, and blood investigations from the medical record of each potentially eligible patient. Study team will use the patient’s initial vital signs, level of consciousness (i.e., first measurement in triage), WBC count, and partial pressure of carbon dioxide (pCO_2_) to calculate qSOFA, SIRS, and EWSs. Blood pressure will be measured by using an electronic sphygmomanometer and results will be recorded in millimeters of mercury (mmHg). MAP will be calculated from SBP and diastolic blood pressure (DBP) using the following equation:

$$MAP=\frac{SBP+2(DBP)}{3}$$

Pulse rate (recorded as beats/min) and oxygen saturation (recorded as a percentage) will be measured using an electronic pulse oximetry device. We will report whether the oxygen saturation reading was in room air or while a patient is on oxygen therapy. Body temperature will be measured orally or (axillary if necessary) by electronic thermometer and recorded as degree Celsius. A new-onset Glasgow Coma Scale (GCS) score of <15 will be considered significant for qSOFA calculation. If it is unclear whether a patient’s confusion is ‘new’ or their usual state, we will assume the altered mental state/confusion is new until confirmed otherwise for all scores calculation.

Initial WBCs count (recorded in x10^9^/μL) and pCO_2_ (recorded in mmHg; if available) will be obtained from the patient’s medical record and entered into the eCRF.

### Sample size

In the PASSEM study, we chose the method suggested by Collins et al [22]. In this method, sample size calculation is based on the expected event rate (minimum of 100 events in all validation datasets). However, rules-of-thumb for sample size are problematic, as they are not specific to the model or validation setting. Indeed, Snell et al showed that the rule-of-thumb of having at least 100 events and 100 non-events does not always produce precise estimates of a model’s predictive performance measures [23]. To overcome this limitation, we chose to target an event rate of ≥200. Previous work by Freund et al [15] showed that a sample size of 879 patients yielded 74 events when power was set at 90%. Therefore, if we target a minimum of 200 events and consider 20% of lost to follow-up and missing data, a sample size of 2851 should be included. We will conduct an interim analysis after recruitment of 25%, 50%, and 75% of the target sample size to re-evaluate our assumptions and correct the sample size accordingly.

### Statistical analysis

Continuous data will be reported as mean (SD) or median (IQR) and compared using unpaired *t* tests or analysis of variance and Mann-Whitney or Kruskal-Wallis test. Categorical variables will be expressed as number (percentage) and compared using a χ^2^ test or a Fisher exact test. We will begin by calculating an overall area under the receiver operating characteristic curve (AUC of ROC curve) and generate calibration curves of the qSOFA, SIRS, and EWSs to predict the primary and secondary outcomes. Subsequent to assessing the model’s overall performance; sensitivity, specificity, positive and negative predictive values will be calculated with cross tables for predicting primary and secondary outcomes for a qSOFA score of ≥2, SIRS of ≥2, and EWSs of ≥5. We will use the Kaplan-Meier method to estimate in-hospital and 90-day all-cause mortality. A log-rank regressions will be used to assess groups’ differences. Odd ratios (ORs) for in-hospital death, ICU admission, and 90-days all-cause mortality of qSOFA, SIRS, and EWSs will be estimated with a logistic regression analysis after adjustment for the patients’ demographics, comorbidities, and CCI. The model fit will be assessed by the calculation of the log-likelihood, Akaike information criterion (AIC), AUC, Bayesian information criterion (BIC), and D-statistics. To compare the performance of qSOFA, SIRS and EWSs, we will use absolute net-reclassification index (NRI). The absolute NRI mathematically represents a net proportion of patients correctly reclassified by one model as compared to another [24]. Net reclassification involves classifying patients in risk categories and determining how a new model reclassifies patients into various risk categories compared with a previous model. Risk differences are classified based on the actual outcome patients experienced (those who died vs those who did not).

A priori subgroup analyses will be conducted based on status of the following: COVID-19 (present vs absent), febrile neutropenia (present vs absent), solid organs or hematological cancers (present vs absent), autoimmune diseases (present vs absent), and severe comorbidities (CCI ≥3 vs <3), and race of the patient (Asian vs Black vs South Asian vs White) as permitted by sample size. If missing data is minimal (<5%) we will conduct a complete case analysis, otherwise we will use multiple imputation.

For all analyses, a 2-tailed *P* <0.05 will be considered statistically significant. Statistical analyses will be performed with Stata version 17.0 [25] and RStudio version ‘2022.7.0.548’ [26].

### Data management

We will use an encrypted, web-based EDC (Castor^®^) for this study [27]. Lead investigators (or their delegates) will enter clinical data on an eCRF at each participating center. They will make all entries, corrections, and alterations. The data manager of this study will provide all tools, instructions, and training necessary to complete the eCRF, and each user will be issued a unique username and password.

The monitors will review the eCRFs, evaluate them for completeness and consistency, and compare them with the source documents to ensure no discrepancies. The Monitors cannot enter data in the eCRFs. Lead investigators must verify that all data entries in the eCRF are accurate and correct. If some assessments are not done, or specific information is unavailable, not applicable, or unknown, they must indicate this in the eCRF. Finally, lead investigators must electronically sign off all patients’ eCRF enrolled from their hospitals.

Data manager will lock the final validated database so that no more change will be possible on the frozen data. Subsequently, the principal investigator will receive the patient data (eCRF data + audit trail) for archiving at the investigational site and transference in a secure way to the biostatistical team in Stata format.

### Ethics

#### Informed consent

Informed consent was waived for this study due to its complete observational nature and absence of interventions or invasive procedures. The study does not impose any change in the standard practice of sepsis at the site; and the patient’s data will be collected prospectively from their medical record at recruiting centers. The benefit/risk ratio of participation in the study is excellent. Moreover, we expect that PASSEM Study results may improve patient care in the recruiting center by allowing a better understanding of ideal tools to identify patients with sepsis.

#### Ethical approval

This protocol complies with the principles laid down by the 59th World Medical Assembly and all applicable amendments laid down by the World Medical Assemblies, the applicable regulations per site, and any other relevant local requirement and laws.

PASSEM study has been approved by local Institution review board (LIRB) of all recruiting hospitals at the time of publication of this protocol. Data manager of PASSEM study will not grant access to the EDC system or start the study until the principal investigator receives a copy of a written and dated approval/favorable signed opinion from each participating center LIRB.

We will present any change in this protocol as an amendment in written form to the protocol. The principal investigator and lead investigators will sign the protocol amendment and then submitted to the LIRBs. Following approval, we will send the amendment to all participating investigators. The amendment cannot be acted upon before the outcome of this decision. However, the study team will submit minor modifications (administrative modifications, including a new recruitment center) to the LIRBs for information purposes only.

### Patient confidentiality

In order to maintain confidentiality, we will not collect any patient’s-identifying data (e.g., name, identification number, medical record number [MRN], etc.) in the eCRF. Instead, lead investigators will store such data in a separate list sheet specified for each participating center. The lead investigator of each center will maintain this list in strict confidence.

## Discussion

PASSEM study will be the first international multicenter prospective cohort study that designated to externally validate qSOFA score, SIRS criteria, and EWSs for in-hospital mortality among adult patients with suspected infection presenting to the ED in the Middle East region. In an ED setting, it is crucial to take a patient’s vital signs as early as possible to make decisions and predict the patient’s outcome. Hence, PASSEM study will use initial physiologic parameters the patient presented with, to the ED (triage vital signs) to calculate qSOFA, SIRS criteria, and EWSs. Furthermore, definition of infection will be based on the opinion of the ED attending physician with subsequent confirmation at the end of in-hospital follow-up, which might be more appropriate and pragmatic.

We will publish study’s results in peer-reviewed journals and may present them at scientific conferences. We will follow recommendations of Transparent Reporting of a Multivariable Prediction Model For Individual Prognosis or Diagnosis (TRIPOD) guidelines [28]. The most significant results will be shared to the public through social networks.

## Supporting information

S1 File (PDF)

## References

[1] Angus DC, van der Poll T. Severe sepsis and septic shock. The New England journal of medicine. 2013;369(9):840–51. Epub 2013/08/30. doi: 10.1056/NEJMra1208623 . 23984731

[2] Cohen J, Vincent JL, Adhikari NK, Machado FR, Angus DC, Calandra T, et al. Sepsis: a roadmap for future research. Lancet Infect Dis. 2015;15(5):581–614. Epub 2015/05/02. doi: 10.1016/S1473-3099(15)70112-X . 25932591

[3] Mouncey PR, Osborn TM, Power GS, Harrison DA, Sadique MZ, Grieve RD, et al. Trial of early, goal-directed resuscitation for septic shock. The New England journal of medicine. 2015;372(14):1301–11. Epub 2015/03/18. doi: 10.1056/NEJMoa1500896 . 25776532

[4] Peake SL, Delaney A, Bailey M, Bellomo R, Cameron PA, Cooper DJ, et al. Goal-directed resuscitation for patients with early septic shock. The New England journal of medicine. 2014;371(16):1496–506. Epub 2014/10/02. doi: 10.1056/NEJMoa1404380 . 25272316

[5] Yealy DM, Kellum JA, Huang DT, Barnato AE, Weissfeld LA, Pike F, et al. A randomized trial of protocol-based care for early septic shock. The New England journal of medicine. 2014;370(18):1683–93. Epub 2014/03/19. doi: 10.1056/NEJMoa1401602 ; PubMed Central PMCID: PMC4101700. 24635773 PMC4101700

[6] Minasyan H. Sepsis and septic shock: Pathogenesis and treatment perspectives. J Crit Care. 2017;40:229–42. Epub 2017/04/28. doi: 10.1016/j.jcrc.2017.04.015 . 28448952

[7] Rhee C, Dantes R, Epstein L, Murphy DJ, Seymour CW, Iwashyna TJ, et al. Incidence and Trends of Sepsis in US Hospitals Using Clinical vs Claims Data, 2009–2014. JAMA. 2017;318(13):1241–9. Epub 2017/09/14. doi: 10.1001/jama.2017.13836 ; PubMed Central PMCID: PMC5710396 28903154 PMC5710396

[8] Sherwin R, Winters ME, Vilke GM, Wardi G. Does Early and Appropriate Antibiotic Administration Improve Mortality in Emergency Department Patients with Severe Sepsis or Septic Shock? The Journal of emergency medicine. 2017;53(4):588–95. Epub 2017/09/17. doi: 10.1016/j.jemermed.2016.12.009 . 28916120

[9] Seymour CW, Liu VX, Iwashyna TJ, Brunkhorst FM, Rea TD, Scherag A, et al. Assessment of Clinical Criteria for Sepsis: For the Third International Consensus Definitions for Sepsis and Septic Shock (Sepsis-3). JAMA. 2016;315(8):762–74. Epub 2016/02/24. doi: 10.1001/jama.2016.0288 ; PubMed Central PMCID: PMC5433435. 26903335 PMC5433435

[10] Shankar-Hari M, Phillips GS, Levy ML, Seymour CW, Liu VX, Deutschman CS, et al. Developing a New Definition and Assessing New Clinical Criteria for Septic Shock: For the Third International Consensus Definitions for Sepsis and Septic Shock (Sepsis-3). JAMA. 2016;315(8):775–87. Epub 2016/02/24. doi: 10.1001/jama.2016.0289 ; PubMed Central PMCID: PMC4910392. 26903336 PMC4910392

[11] Singer M, Deutschman CS, Seymour CW, Shankar-Hari M, Annane D, Bauer M, et al. The Third International Consensus Definitions for Sepsis and Septic Shock (Sepsis-3). JAMA. 2016;315(8):801–10. Epub 2016/02/24. doi: 10.1001/jama.2016.0287 ; PubMed Central PMCID: PMC4968574. 26903338 PMC4968574

[12] Gauer R, Forbes D, Boyer N. Sepsis: Diagnosis and Management. American family physician. 2020;101(7):409–18. Epub 2020/04/02. . 32227831

[13] Sabir L, Ramlakhan S, Goodacre S. Comparison of qSOFA and Hospital Early Warning Scores for prognosis in suspected sepsis in emergency department patients: a systematic review. Emerg Med J. 2021. Epub 2021/08/19. doi: 10.1136/emermed-2020-210416 . 34404680

[14] Evans L, Rhodes A, Alhazzani W, Antonelli M, Coopersmith CM, French C, et al. Surviving sepsis campaign: international guidelines for management of sepsis and septic shock 2021. Intensive Care Med. 2021;47(11):1181–247. Epub 2021/10/03. doi: 10.1007/s00134-021-06506-y ; PubMed Central PMCID: PMC8486643. 34599691 PMC8486643

[15] Freund Y, Lemachatti N, Krastinova E, Van Laer M, Claessens YE, Avondo A, et al. Prognostic Accuracy of Sepsis-3 Criteria for In-Hospital Mortality Among Patients With Suspected Infection Presenting to the Emergency Department. JAMA. 2017;317(3):301–8. Epub 2017/01/24. doi: 10.1001/jama.2016.20329 . 28114554

[16] Haydar S, Spanier M, Weems P, Wood S, Strout T. Comparison of QSOFA score and SIRS criteria as screening mechanisms for emergency department sepsis. The American journal of emergency medicine. 2017;35(11):1730–3. Epub 2017/07/18. doi: 10.1016/j.ajem.2017.07.001 . 28712645

[17] Hwang SY, Jo IJ, Lee SU, Lee TR, Yoon H, Cha WC, et al. Low Accuracy of Positive qSOFA Criteria for Predicting 28-Day Mortality in Critically Ill Septic Patients During the Early Period After Emergency Department Presentation. Annals of emergency medicine. 2018;71(1):1–9.e2. Epub 2017/07/04. doi: 10.1016/j.annemergmed.2017.05.022 . 28669551

[18] Churpek MM, Snyder A, Han X, Sokol S, Pettit N, Howell MD, et al. Quick Sepsis-related Organ Failure Assessment, Systemic Inflammatory Response Syndrome, and Early Warning Scores for Detecting Clinical Deterioration in Infected Patients outside the Intensive Care Unit. Am J Respir Crit Care Med. 2017;195(7):906–11. Epub 2016/09/21. doi: 10.1164/rccm.201604-0854OC ; PubMed Central PMCID: PMC5387705. 27649072 PMC5387705

[19] Goulden R, Hoyle MC, Monis J, Railton D, Riley V, Martin P, et al. qSOFA, SIRS and NEWS for predicting inhospital mortality and ICU admission in emergency admissions treated as sepsis. Emerg Med J. 2018;35(6):345–9. Epub 2018/02/23. doi: 10.1136/emermed-2017-207120 . 29467173

[20] Nieves Ortega R, Rosin C, Bingisser R, Nickel CH. Clinical Scores and Formal Triage for Screening of Sepsis and Adverse Outcomes on Arrival in an Emergency Department All-Comer Cohort. The Journal of emergency medicine. 2019;57(4):453–60.e2. Epub 2019/09/11. doi: 10.1016/j.jemermed.2019.06.036 . 31500993

[21] Usman OA, Usman AA, Ward MA. Comparison of SIRS, qSOFA, and NEWS for the early identification of sepsis in the Emergency Department. The American journal of emergency medicine. 2019;37(8):1490–7. Epub 2018/11/25. doi: 10.1016/j.ajem.2018.10.058 . 30470600

[22] Collins GS, Ogundimu EO, Altman DG. Sample size considerations for the external validation of a multivariable prognostic model: a resampling study. Stat Med. 2016;35(2):214–26. Epub 2015/11/11. doi: 10.1002/sim.6787 ; PubMed Central PMCID: PMC4738418. 26553135 PMC4738418

[23] Archer L, Snell KIE, Ensor J, Hudda MT, Collins GS, Riley RD. Minimum sample size for external validation of a clinical prediction model with a continuous outcome. Stat Med. 2021;40(1):133–46. Epub 2020/11/06. doi: 10.1002/sim.8766 . 33150684

[24] Alba A, Agoritsas T, Walsh M, et al. Discrimination and calibration of clinical prediction models: Users’ guides to the medical literature. JAMA. 2017;318(14):1377–84. doi: 10.1001/jama.2017.12126 29049590

[25] StataCorp. Stata Statistical Software: Release 17. 17.0 ed. College Station, TX: StataCorp LLC; 2021.

[26] Team R. RStudio: Integrated Development Environment for R. 2022.7.0.548 ed. Boston, MA: RStudio, PBC; 2022.

[27] Castor EDC. Castor Electronic Data Capture Netherlands2021 [24 October 2021]. Available from: https://castoredc.com.

[28] Collins GS, Reitsma JB, Altman DG, Moons KGM. Transparent reporting of a multivariable prediction model for individual prognosis or diagnosis (TRIPOD): the TRIPOD statement. BMJ: British Medical Journal. 2015;350. doi: 10.1136/bmj.g7594 25569120

