# Supplementary material for: Prognostic accuracy of qSOFA score, SIRS criteria, and EWSs for in-hospital mortality among adult patients presenting with suspected infection to the emergency department (PASSEM) Multicenter prospective external validation cohort study protocol
Source: PLoS One. 2024 Jan 17;19(1):e0281208. doi: 10.1371/journal.pone.0281208 (PMC10793907; doi:10.1371/journal.pone.0281208)
Supplement: S1 File — (PDF) [file pone.0281208.s001.pdf]

Prognostic Accuracy of qSOFA, SIRS, and EWSs in ED setting (PASSEM)

Case report form

Study ID: ____________________

Screening

Eligibility screening

1.1. Date of birth (Gregorian): __ /__ /____ (DD/MM/YYYY)

Exclude if age <18 years

1.2. Sex

- ☐ Men (1)
- ☐ Women (2)

1.3. Pregnant ☐ Yes (1) ☐ No (0)

Exclude if pregnant

1.4. Patient transferred from another hospital? ☐ Yes (1) ☐ No (0)

Exclude if transferred from another hospital

1.5. Presentation to ED mostly due to infection? ☐ Yes (1) ☐ No (0)

Exclude if presentation to ER was not due to infection

- **What is the initial Dx in ER?** ____

1.6. Patient will be admitted to the hospital? ☐ Yes (1) ☐ No (0)

Exclude if patient discharged from ED

1.7. Patient admitted to the hospital through ED? ☐ Yes (1) ☐ No (0)

Exclude if patient electively admitted to the hospital

1.8. Code status of patient is "Do-Not-Resuscitate" (DNR)? ☐ Yes (1) ☐ No (0)

Exclude if patient's code status is "DNR"

1.9. Informed consent obtained? ☐ Yes (1) ☐ No (0)

Exclude if patient refused to participate in the study (ONLY if recruiting center required informed consent)

Enrolment

Demographics

2.1.1. Inclusion date: __ /__ /____ (DD/MM/YYYY)

2.1.2. Race

- ☐ Asian (1)
- ☐ Black (2)
- ☐ South Asian (3)
- ☐ White (4)

History

2.2.1. Patient known case of chronic respiratory diseases (e.g., asthma, COPD, ILD)? ☐ Yes (1) ☐ No (0)

2.2.2. Patient known case of myocardial infarction? ☐ Yes (1) ☐ No (0)

2.2.3. Patient known case of dementia? ☐ Yes (1) ☐ No (0)

2.2.4. Patient known case of heart failure? ☐ Yes (1) ☐ No (0)

2.2.5. Patient known case of peripheral vascular disease? ☐ Yes (1) ☐ No (0)

2.2.6. Patient known case of stroke or TIA? ☐ Yes (1) ☐ No (0)

2.2.7. Patient known case of rheumatological or autoimmune diseases? ☐ Yes (1) ☐ No (0)

2.2.8. Patient known case of peptic ulcer disease? ☐ Yes (1) ☐ No (0)

2.2.9. Patient known case of liver disease?
- ☐ No (0)
- ☐ Mild (1)
- ☐ Moderate to severe (2)

2.2.10. Patient known case of diabetes mellitus?
- ☐ None (0)
- ☐ Diet-controlled (1)
- ☐ Uncomplicated (2)
- ☐ End-organ damage (3)

2.2.11. Patient known case of hypertension or using hypertension medications? ☐ Yes (1) ☐ No (0)

2.2.12. History of smoking? ☐ Yes (1) ☐ No (0)

2.2.13. Patient known case of hemiplegia/paraplegia? ☐ Yes (1) ☐ No (0)

2.2.14. Patient known case of chronic kidney disease (CKD)? ☐ Yes (1) ☐ No (0)

2.2.15. Patient known case of cancer (solid tumor)?
- ☐ No (0)
- ☐ Localized (1)
- ☐ Metastatic (2)

2.2.16. Patient known case of leukemia? ☐ Yes (1) ☐ No (0)

2.2.17. Patient known case of lymphoma? ☐ Yes (1) ☐ No (0)

2.2.18. Patient known case of AIDS? ☐ Yes (1) ☐ No (0)

Vital signs and blood tests

2.3.1. Altered mental status (GCS <15)? ☐ Yes (1) ☐ No (0)

2.3.2. AVPU Score?

☐ Alert (0)
☐ Reacts to voice (1)
☐ Reacts to pain (2)
☐ Unresponsive (3)

2.3.3. Hypercapnic respiratory failure ($PaCO_2$ >45 mmHg ± pH <7.35)? ☐ Yes (1) ☐ No (0)

2.3.4. Body temperature: ______ °C

2.3.5. Heart rate: ______ beats/minute

2.3.6. Systolic blood pressure (SBP): ______ mmHg

2.3.7. Diastolic blood pressure (DBP): ______ mmHg

2.3.8. Respiratory rate: ______ breaths/minute

2.3.9. Oxygen saturation: ______ %

2.3.10. Any supplemental oxygen? ☐ Yes (1) ☐ No (0)

2.3.11. White blood cell (WBC) count: ______ $\times 10^3/\mu L$

In-hospital follow-up (30-days)

Infection status

3.1.1. Was the patient discontinued from the study? ☐ Yes (1) ☐ No (0)

If yes to question 3.1.1.:

- **Date of discontinuation:** __ / __ / ____ (DD/MM/YYYY)
- **Reason(s) for discontinuation:** ______________________________

3.1.2. Was the initial presentation to ED due to infection? ☐ Yes (1) ☐ No (0)

Exclude if initial presentation was not due to infectious cause

3.1.3. If yes, infection confirmed by:

☐ Positive body fluid(s) culture (1)
☐ Other microbiological methods (e.g., serology, PCR, ... etc.) (2)
☐ Radiological evidence (3)
☐ Clinical context (4)

3.1.4. If yes to 3.1.2.: What was the source of infection?

☐ COVID-19 (1)
☐ Respiratory (except COVID-19) (2)

☐ Urinary (3)
☐ Abdominal (4)
☐ Soft tissue/cutaneous (5)
☐ Neurological (6)
☐ Bone and joints (7)
☐ Bloodstream (8)
☐ Unknown (9)
☐ Other (10), specify please: ____

In-hospital mortality (30-days)

3.2.1. What is the status of patient by end of in-hospital follow-up?

☐ Died (1)
☐ Discharged from hospital (2)
☐ Transferred to another hospital (3)
☐ Still hospitalized (4)

3.2.2. If died, what was the cause of death?

☐ COVID-19 (1)
☐ Heart ds (MI, CHF, dysrhythmia, ... etc.) (2)
☐ Respiratory ds (COPD, BA, respiratory failure, ... etc.) (3)
☐ Sepsis-related (septic shock, MODS, ... etc.) (4)
☐ Cancer (Solid or hematological) (5)
☐ Stroke (including intracranial hemorrhage) (6)
☐ Other (7), specify please: ____

3.2.3. If died, specify date of death: __ / __ / ____ (DD/MM/YYYY)

3.2.4. If discharged from hospital, specify date of discharge: __ / __ / ____ (DD/MM/YYYY)

3.2.5. If transferred to another hospital, specify date of transfer: __ / __ / ____ (DD/MM/YYYY)

ICU-admission (30-days)

3.3.1. Has patient admitted to ICU? ☐ Yes (1) ☐ No (0)

- **If admitted to ICU, specify date of ICU admission:** __ / __ / ____ (DD/MM/YYYY)

3.3.2. Has patient discharged from ICU? ☐ Yes (1) ☐ No (0)

- **If discharged from ICU, specify date of discharge from ICU:** __ / __ / ____ (DD/MM/YYYY)

Out-hospital follow-up (90-days)

All-cause mortality (90-days)

4.1. What is the status of patient by end of in-hospital follow-up?

☐ Died (1)
☐ Alive (2)

4.2. If died, what was the cause of death?

☐ COVID-19 (1)

- ☐ Heart ds (MI, CHF, dysrhythmia, ... etc.) (2)
- ☐ Respiratory ds (COPD, BA, respiratory failure, ... etc.) (3)
- ☐ Sepsis-related (septic shock, MODS, ... etc.) (4)
- ☐ Cancer (Solid or hematological) (5)
- ☐ Stroke (including intracranial hemorrhage) (6)
- ☐ Other (7); specify please: ____

4.3. If died, specify date of death: __ / __ / ____ (DD/MM/YYYY)
