# Supplementary material for: Prognostic accuracy of qSOFA score, SIRS criteria, and EWSs for in-hospital mortality among adult patients presenting with suspected infection to the emergency department (PASSEM) Multicenter prospective external validation cohort study protocol
Source: PLoS One. 2024 Jan 17;19(1):e0281208. doi: 10.1371/journal.pone.0281208 (PMC10793907; doi:10.1371/journal.pone.0281208)
Supplement: Attachment — Submitted filename: renamed_01d0c.pdf [file pone.0281208.s002.pdf]

PASSEM
Sepsis in Emergency Department Cohort Study

Dear Editors,

Thank you for the opportunity to address the comments from the Dr. Narani Sivayoham. The authors hope that the Reviewer and Editors will be satisfied with the further amendments which we have made to the manuscript.

We believe that the manuscript is now suitable for publication in PLOS ONE.

Dr. Abdullah M. Algarni
(ACH) Family Medicine Senior Registrar

Abdullah M. Algarni, MD
Family medicine consultant
Aseer Central Hospital
Abha, Saudi Arabia

On behalf of all authors

REVIEWER #1:

Thank you for the opportunity to review this study protocol entitled, 'Prognostic accuracy of qSOFA score, SIRS criteria and EWSs for in-hospital mortality among adult patients presenting with suspected infection to the emergency department (PASSEM): Multicenter prospective external validation cohort study protocol'. In this prospective multicenter observational study, the authors plan to validate multiple scores to study their prognostic accuracy for 30-day mortality in patients with an infection. The protocol is well written and reasonably easy to follow. The authors should be commended for undertaking such a large multicenter study.

However, I do have some minor concerns. They are as follows:

1. Primary end-point

The abstract states '30 day in-hospital mortality'. Not all patients are admitted for 30 days. So, I would expect the primary end-point to be either 30-day mortality or in-hospital mortality. Please clarify the primary end-point.

1.1. Authors response

Thank you. We have anticipated that issue by using Kaplan-Meier survival estimate. Hence, participants who didn't die within 30 days will be censored. Moreover, we have two distinct death-related outcomes within a 30-day time frame: the first is the study's primary outcome, which is in-hospital mortality, and the second is all-cause mortality (in/out-hospital mortality).

2. Primary and secondary end-points

Please state the primary and secondary end points at the end of the introduction.

2.1. Authors response

Thank you, we have adjusted accordingly.

3. Hypothesis

The authors have hypothesized that qSOFA has greater prognostic accuracy. It is usual to have a null-hypothesis and the result should either maintain or reject the null-hypothesis. Could this be reworded as such?

3.1. Authors response

Thank you, we have adjusted accordingly.

4. Partial pressure of carbon dioxide

The authors have stated that they will be noting the partial pressure of carbon dioxide. Does this mean all patients will have an arterial blood gas? What is the purpose? Is it to determine if the patient is a CO_2 retainer? If so, is this parameter only for those with COPD?

4.1. Authors response

Thank you; it will be recorded if it is done as part of patient emergency care, but the protocol does not mandate if it is unavailable. Moreover, most emergency departments in the Gulf region use venous blood gases (VBG), not arterial ones (ABG), as a metabolic screen on all critical illnesses (ABG only if VBG shows acute respiratory acidosis, patient considered for non-invasive respiratory support [NIV], high-flow nasal cannula [HFNC], or invasive positive pressure ventilation [IPPV]).

5. Follow-up

The authors have stated that ‘the study does not impose any change in the standard practice’. The protocol also states, ‘we will determine their status via telephone contact’. Is it standard practice to telephone patients to determine their status? Do patient not need to consent to receiving a telephone call?

5.1. Authors response

Thank you; we agree that calling patients for follow-up is not the usual care after a while. However, most participating hospitals will determine the patients' status by reviewing the mortuary database linked to the national death registry. On less often occasions where such linking is not present, consent will be obtained to follow the patient's status. Moreover, the protocol is supported by all participating hospitals' ethical approval boards and committees.
